# Vaccine uptake and associated factors in an irregular urban settlement in northeastern Brazil: a cross-sectional study

**DOI:** 10.1186/s12889-020-09247-7

**Published:** 2020-07-22

**Authors:** Ana Amélia Corrêa de Araújo Veras, Eduardo Jorge da Fonseca Lima, Maria de Fátima Costa Caminha, Suzana Lins da Silva, Amanda Alves Moreira de Castro, Andressa Lílian Bezerra Bernardo, Maria Lídia Amaral Barbosa Ventura, Pedro Israel Cabral de Lira, Malaquias Batista Filho

**Affiliations:** 1 Instituto de Medicina Integral Professor Fernando Figueira, Rua dos Coelhos, 300 - Boa Vista, Recife – PE. CEP, Recife, Pernambuco 50070-902 Brazil; 2 Faculdade Pernambucana de Saúde, Avenida Mal, Avenida Mal. Mascarenhas de Morais, 4861 - Imbiribeira, Recife-PE. CEP, Recife, Pernambuco 51150-000 Brazil; 3 Universidade Federal de Pernambuco, Av. Prof. Moraes Rego, 1235 - Cidade Universitária, Recife - PE, Recife, Pernambuco 50670-901 Brazil

**Keywords:** Children’s health, Vaccination schedule, Maternal education level, Family health strategy

## Abstract

**Background:**

Globally, childhood immunization saves the lives of 2–3 million children annually by protecting them against vaccine-preventable diseases. In 2017, 116.2 million children were vaccinated worldwide according to the World Health Organization. Nevertheless, figures suggest that 19.5 million children around the world fail to receive the benefits of complete immunization.

**Methods:**

This cross-sectional study analyzed vaccine uptake and the factors associated with incomplete vaccination schedule in children of up to 36 months of age assisted by the family health strategy in an irregular settlement located in a state capital city in northeastern Brazil. This study was nested within a larger study entitled “Health, nutrition and healthcare services in an urban slum population in Recife, Pernambuco”, conducted in 2015. A census included 309 children, with vaccination data obtained, exclusively, from their vaccination cards records. An ad hoc database was constructed with variables of interest. Absolute and relative values were calculated for the socioeconomic, demographic, obstetric and biological data. To identify possible factors associated with incomplete vaccination schedule, crude and multivariable Poisson regression analyses were performed, and conducted in accordance with the forward selection method with robust variance and the adjusted prevalence ratio was calculated with the 95% CI. Variables with *p*-values < 0.20 in the unadjusted stage were included in the multivariable analysis. The statistical significance of each variable was evaluated using the Wald test, with *p*-values < 0.05.

**Results:**

Just half of the children (52,1%) was classified as complete vaccination schedule. In the final model, the factors associated with incomplete vaccination schedule were age 12–36 months and the mother who did not complete high school.

**Conclusion:**

The percentage of vaccine uptake found was far below the recommendation of the National Childhood Immunization Schedule and was associated with child’s age and mother’s education level. Based on these findings, the family healthcare teams may elaborate vaccination strategies aimed at reaching the coverage rates established by the national immunization program. Optimizing coverage will ultimately prevent the resurgence, at epidemic level, of infectious diseases that are already under control in this country.

## Background

Globally, childhood immunization is considered one of the most effective health interventions, playing a significant role in reducing child mortality and saving the lives of 2–3 million children annually by protecting them against vaccine-preventable diseases [1]. In 2017, 116.2 million children were vaccinated worldwide according to the World Health Organization [2]. Nevertheless, the United Nations International Children’s Emergency Fund (UNICEF) estimated that 19.5 million children around the world had not been given the benefits of complete immunization [1].

The countries of Latin America have made significant progress in immunization, both in terms of including new vaccines in their schedules and of increasing vaccination coverage. According to UNICEF, vaccine uptake is low among children of poor families in the world and it is estimated that one in every five children fails to receive the basic immunization required for health and survival [1]. In countries with different development levels, the complete vaccination schedule could be affected by factors related to culture and geographical localization, and has been associated with later birth order, with poorer maternal education levels and with socioeconomic conditions [3–5].

Other factors reported to influence the vaccine uptake include the sex and age of the child, the number of children in the home, the number of prenatal visits, the month in which prenatal care is initiated, care at childbirth, where the child is born, and the mother’s anti-tetanus vaccination status [6–9].

A survey conducted in the Brazilian state capital cities showed better immunization coverage in children from low-income families [6]. However, another study reported higher rates of incomplete immunization with new vaccines in children from low social classes D and E (Additional file 1) [7]. Recently a cohort study in the south Brazilian region, revealed high family income as associated factor with incompleteness coverage, highlighting differences within the country in relation to income and settings [10].

In the last decade, other questions have been raised regarding the complete vaccination schedule, including the habits and beliefs of the parents with regard to vaccination [11], concerns with safety in relation to possible adverse events of the vaccines [12], hesitation as for immunization [13], and parents/guardians interrupting vaccination or even refusing to vaccinate their children [11], partially as a result of campaigns on the Internet and on social networks led by anti-vaccine groups and movements [14].

Some characteristics inherent to healthcare services and to work procedures also interfere with vaccine uptake and include: difficulty with access and cost [12], inadequate stocks of immunobiological agents, discordant information given to the parents by the healthcare professionals [11], opportunities missed when the child attends the healthcare service for a consultation, failing to review the immunization record in the healthcare service, and the establishment of fixed dates for some vaccinations [15].

When vaccine coverage in Brazil was calculated based on data obtained from the immunization schedule evaluation database (SI-API), which consolidates data obtained at all management levels, it is important to emphasize that this does not permit evaluation of the vaccination schedule, individually. The data available provide the vaccination rate for a given immunobiological agent, which is calculated from the total number of doses used and the estimated calculation of the target population [16].

Hence, biases may be present in data recording or even in the calculation of the size of the target population. Furthermore, with the differences in coverage across the country’s population, which are not always perceptible through these indicators, there is a risk of overlooking large pockets of susceptible individuals capable of determining the introduction and/or maintenance of the circulation of infectious agents [17].

Epidemiological household surveys are considered the most reliable means of monitoring vaccination coverage, since the doses of vaccine received are verified directly from the child’s immunization record card. The recommendation is that this verification be performed every 3 to 5 years [18].

In this perspective, the present study assessed the completeness of the Brazilian vaccination schedule and the factors associated in children of up to 36 months of age registered in the Family Health Strategy in an irregular settlement area which is located in a state capital city in northeastern Brazil. The objective was to provide evidence to support the development of strategies by family healthcare teams to successfully reach unvaccinated or partially vaccinated children.

## Methods

### Study design and population

This was a cross-sectional study nested within a larger survey entitled “Health, nutrition and healthcare services in an urban slum population in Recife, Pernambuco”. Data was collected from children’s vaccination cards over a four-month period from July to October 2015 The sample, based on a community census, included 310 children living in an irregular settlement in Recife, the state capital of Pernambuco in northeastern Brazil. A primary healthcare unit was inaugurated in this community of 7400 residents in 1990, and, from 2000 onwards, two family health units were in operation with similar teams and work processes, including strategies to call/recall for vaccination. The children were identified from records kept by the community health agents and from the patient charts at the family health units. Children of up to 36 months of age and for whom data on immunization were available through the child’s vaccination card were included in the study. Only one child did not present vaccination records on the card, thus being excluded, resulting in a final sample of 309 children.

### Ethics

The data for the original study were obtained by interviewing the child’s mother or guardian after he/she had been invited to participate in the study and had signed an informed consent form. The internal review board of the *Instituto de Medicina Integral Professor Fernando Figueira* (IMIP) approved the study protocol under reference CAEE: 80877817.6.0000.5201.

### Outcome

The outcome variable was vaccination schedule status, categorized as complete if the child had received the total number of doses of each vaccine scheme for age in accordance with the 2015 National Childhood Immunization Schedule of the Brazilian Ministry of Health (Additional file 1), otherwise as incomplete if the doses of the vaccine had not been administered within 30 days of the established date for all age groups, as referenced in a similar study [19]. Data on immunization were collected, exclusively, from the child’s vaccination card records, shown by all participating children to the interviewers in the original research.

### Explanatory variables

Based on the original study, an ad hoc database was constructed in which the variables of specific interest to this study were recorded. The structured questionnaire used to collect data included questions on socioeconomic and environmental conditions and child care. Social class, community housing for 10 years or more, type of dwelling, mother’s age, mother completed high school, mother received prenatal care, number of prenatal consultation, prenatal care started in the first trimester of pregnancy, child’s gender and child’s age group were included as explanatory variables. Social class was collected through a structured and standardized questionnaire including variables such as home conditions, possession of goods (automobiles, computers and others), head of household education and access to public services (running water and paved street) and classified as A, B1, B2, C1, C2 and D-E according with a punctuation obtained in Brazil Criterion (see Additional file 2). Type of dwelling was researched as a house or other types of housing (stilt house/hut/single room). Mother’s age in complete years was classified in < 20, 20 to 35, and ≥ 36, number of prenatal consultations, from mothers’ verbal reports was categorized as 0 to 5 and 6 or more, according to the Brazilian Ministry of Health. Community housing for 10 years or more, mother completed high school, mother received prenatal care, prenatal care started in the first trimester of pregnancy were classified as yes/no. Child’s gender was categorized as male and female and child’s age was collected individually and then organized into three age groups: 0 to < 6, 6 to < 12 and 12 to 36 months.

### Statistical analyses

Data analysis was performed using the Stata software program, version 12.1. Absolute and relative values were calculated for the population evaluated in relation to the socioeconomic variables (social class, type of residence, time living in the community), the mother’s demographic data (age and whether she had completed high school), obstetric variables (whether the woman had received prenatal care, whether prenatal care was initiated in the first trimester of pregnancy, and the number of prenatal consultations) and the child’s biological variables (sex and age). To identify the possible factors associated with completeness of the vaccination schedule, univariate Poisson regression *with robust standard errors* was initially used to calculate the crude prevalence ratio and respective 95% confidence intervals (95%CI). The variables resulting in *p*-values < 0.20 at this stage were then included in a multivariate Poisson regression analysis. It was conducted in accordance with the forward selection method with robust variance and the adjusted prevalence ratio was calculated together with the 95%CI. The statistical significance of each variable was evaluated using the Wald test, with *p*-values < 0.05.

## Results

A total of 309 children were included in the study. Of these, 111 (35.9%) were registered with family health unit I, while 198 (64.1%) were registered with family health unit II.

Only 52.1% of the children were found to have complete vaccination schedule status, as recommended by the National Immunization Program (Table 1). A gradual reduction was found in the percentage of expected results (specific quantitative goals established by the National Immunization Program). These goals were met by 83.6% of the children of 0 to < 6 months of age, by 68.3% of those of 6 to < 12 months of age, and, finally, by 36.9% of children of 12 to 36 months of age. Completeness was inversely and significantly associated with age (*p* <  0.001).

**Table 1:** Vaccine uptake in children aged 0 to 36 months. Recife, Pernambuco, Brazil, 2015

Age Group (months)	Vaccination schedule status		***p***-value
	Complete	Incomplete	
	n (%)	n (%)	< 0.001
0 to < 6	**61 (83.6)**	12 (16.4)	
6 to < 12	28 (68.3)	13 (31.7)	
12 to 36	**72 (36.9)**	123 (63.1)	
**Total**	**161 (52.1)**	**148 (47.9)**	

Complete vaccination schedule status was defined if the child has reached the complete scheduled vaccine doses of National Childhood Immunization Schedule of the Brazilian Ministry of Health recommended for each age (months) in 2015 otherwise as incomplete if the doses of the vaccine had not been administered up to 30 days of the established date (see Additional file 1)

Table 2 shows the socioeconomic, demographic, maternal, obstetric and biological data for these children. For 63.4% of the families, total income was less than R$1277.00, the equivalent of 1.6 minimum salaries in 2015. Most of the families had lived in the community for more than 10 years and the majority lived in a house. Around one-fifth of the population lived in precarious dwellings such as stilt houses, huts or single rooms.

**Table 2:** Socioeconomic, demographic, obstetric and biological characteristics of children aged 0 to 36 months. Recife, Pernambuco, Brazil, 2015

Characteristics	n (%)
**Social class**^a^	
A	–
B1 or B2	15 (4.8)
C1 or C2	224 (72.5)
D or E	**70 (22.6)**
**Community housing for ten years or more**	
Yes	**242 (78.3)**
No	67 (21.7)
**Type of dwelling**	
House	247 (79.9)
Stilt house/hut/single room	**62 (20.1)**
**Maternal age (years)**	
< 20	51 (16.5)
20–35	**229 (74.1)**
≥ 36	29 (9.4)
**Mother completed high school**^b^	
Yes	111 (36.2)
No	**196 (63.8)**
**Mother received prenatal care**^b^	
Yes	**285 (92.5)**
No	23 (7.5)
**Number of prenatal consultations**^b^	
1–5	53 (19.2)
≥ 6	**223 (80.8)**
**Prenatal care started in the first trimester**^b^	
Yes	**198 (71.0)**
No	81 (29.0)
**Child’s gender**	
Male	155 (50.2)
Female	154 (49.8)
**Child’s age group (months)**	
0 to < 6	73 (23.6)
6 to < 12	41 (13,3)
12 to 36	195 (63,1)

^a^ Socioeconomic class according to the 2014 definitions established by the Brazilian Market Research Association (ABEP) (see Additional file 2). ^b^ Sample size varies due to missing data

Most of the mothers (74.1%) were between 20 and 35 years of age and 63.8% had failed to complete high school. The majority (92.5%) had received antenatal care, with 70.1% having started prenatal care in the first trimester of pregnancy and 80.8% having attended six or more prenatal sessions. The sample consisted of similar numbers of male and female children. Regard to the child group of age, the highest percentage was 12 to 36 months.

The results of the crude and adjusted analyses regarding completeness of the vaccination schedule are shown in Table 3 together with the exploratory variables. In the final model, the factors associated with incomplete vaccination schedule were age between 12 and 36 months (*p* <  0.001) and the mother not having completed high school (*p* = 0.017).

**Table 3:** Factors influencing vaccine uptake in children aged 0 to 36 months. Recife, Pernambuco, Brazil, 2015

Factors	Sample ***n*** = 309^**a**^	Incomplete vaccination schedule				
		n (%)	Crude PR ^**b**^ (95%CI)	***p***-value ^**c**^	Adjusted PR ^**b**^ (95%CI)	***p***-value
**Social class**^**d**^				0.830		–
A	–				–	
B1 or B2	(*n* = 15)	7 (46.7)	0.88 (0.50–1.53)		**–**	
C1 or C2	(*n* = 224)	119 (53.1)	1			
D or E	(*n* = 70)	35 (50.0)	0.94 (0.72–1.23)			
**Community housing for ten years or more**				0.804		
≥10 years	(*n* = 242)	127 (52.5)	1			
< 10 years	(*n* = 67)	34 (50.7)	0.97 (0.74–1.26)			
**Type of dwelling**				0.247		–
House	(*n* = 247)	133 (53.8)	1		–	
Stilt house/hut/single room	(*n* = 62)	28 (45.2)	0.84 (0.62–1.13)		–	
**Maternal age (years)**				0.299		–
< 20*	(*n* = 51)	31 (60.8)	1		–	
20–35*	(*n* = 229)	114 (49.8)	0.82 (0.63–1.06)		–	
> 36*	(*n* = 29)	16 (55.2)	0.91 (0.61–1.35)		**–**	
**Mother completed high school**				**0.017**		**0.006**
Yes	(*n* = 111)	68 (61.3)	1		1	
No	(*n* = 196)	93 (47.4)	**0.77 (0.63–0.95)**		**0.76 (0.63–0.92)**	
**Number of prenatal consultations**				0.517		–
1–5	(*n* = 53)	27 (50.9)	0.91 (0.68–1.21)		–	
≥6	(*n* = 223)	125 (56.0)	1		–	
**Prenatal care started in the first trimester**				0.710		–
Yes	(*n* = 198)	110 (55.6)	1		–	
No	(*n* = 81)	43 (53.1)	0.95 (0.75–1.21)		–	
**Child’s gender**				0.862		–
Male	(*n* = 155)	80 (51.6)	0.98 (0.79–1.22)		–	
Female	(*n* = 154)	81 (52.6)	1		–	
**Child’s age group (months)**				**< 0.001**		**< 0.001**
0 to < 6	(*n* = 73)	61 (83.6)	1		1	
6 to < 12	(*n* = 41)	**28 (68.3)**	**0.82 (0.65–1.03)**		**0.82 (0.65–1.03)**	
12 to 36	(*n* = 195)	**72 (36.9)**	**0.44 (0.36–0.54)**		**0.44 (0.36–0.54)**	

^a^ Sample size varies due to missing data. ^b^ Prevalence ratio. ^c^ Wald test. ^d^ Economic class according to the Brazilian Market Research Association (ABEP) 2014 definitions (Estimated monthly household income in Brazilian reais: B1 = R$6006.00, B2 = R$3118.00; C1 = R$1865.00, C2 = R$1277.00; D/E = R$895.00) (see Additional file 2)

## Discussion

Completeness of the vaccination schedule in the population evaluated in the present study was 52.1%, far below the goal of 95% established by the National Immunization Program and also below the coverage achieved in infants under 12 months of age in Recife in 2015 with respect to the third dose of the diphtheria-tetanus-acellular pertussis-hepatitis B virus-inactivated poliovirus and *Haemophilus influenzae* type B (pentavalent DTaP-HB-IPV-Hib) vaccine [20].

The pentavalent vaccine is considered a good indicator of the complete vaccination schedule, since it reflects the capacity of the healthcare service to reach the same child and deliver the series of three doses required [21]. The percentage of coverage found in this community is comparable to statistics for countries classified as having medium to low human development indices such as Pakistan, India and Ethiopia. In those countries, the rates of complete vaccine coverage for children of 12 to 23 months of age were 51.3% [8], 53% [9] and 58.4% [22], respectively.

Possible interruptions in the supply of immunobiological agents in healthcare services, due to brief shortages, particularly with respect to the vaccines most recently included in the immunization schedule, could have interfered with the results obtained, as found in a study conducted to analyze the coverage of new vaccines offered in the vaccination schedule [7]. The restriction considering the child’s age in months and days for application of the oral vaccine against human rotavirus, justified for safety reasons, may also constitute a relevant factor.

The significant reduction in the incidence of vaccine-preventable diseases in recent decades could have changed parents’ perceptions regarding the benefits of vaccinating children in relation to potential adverse events, despite the fact that these are rare [5]. Missing the opportunity to vaccinate children attending a healthcare service for another reason or even when they are just accompanying their mothers also contributes to the low rates of immunization coverage [15].

An inverse association was found in the present study between completeness of the vaccination schedule and the child’s age. This finding is in agreement with the results of a prospective cohort study conducted in the Brazilian state of Maranhão in which incomplete vaccination increased as a function of the child’s age [11]. A study conducted with hospitalized children in Recife reported similar findings [23]. This could be attributed to the fact that most of the vaccines included in the vaccination schedule are given in the first year of life, especially up to 6 months, on dates that match with the child’s routine check-up visit. We emphasize that the probability of having a lesser delay in children under 6 months may have been influenced by the greater number of outpatient visits for clinical monitoring during this period when compared with older children, which may have led to some classification bias [26}. After this age, monitoring at the healthcare unit becomes less frequent and the risk of incomplete vaccination schedule increases in the older age group. If so, parents’ attention may be diverted to a younger sibling [5, 22].

Nonetheless, in communities in which the Family Health Strategy is in operation, completeness of the vaccination schedule should not be linked exclusively to the child going to the healthcare unit, but should also be associated with monthly home visits by community health agents as part of their attributions in promoting health within their defined geographical area [24]. Another family health strategy activity that encourages vaccination is actively searching for children who have failed to attend for their vaccination on the scheduled date by ensuring that the control card in the vaccination room is correctly filed and used within an appropriate timeframe and in a programmed manner [16].

The finding of an association between complete vaccination schedule and maternal education level corroborates another reports [3, 5, 7, 9, 23]. The association between poor maternal schooling and incomplete vaccination can be explained by the fact that education level influences knowledge on the different types of vaccine, why they are necessary, their availability, recommendations, benefits and risks [9]. This finding could be used to assist the health care team in identifying and monitoring more closely the children of mothers with low schooling in order to implement their vaccination. It signals that this mother needs to receive more attention from them by being available and providing clarification on the importance of updating vaccines in a simpler and understandable way for her. It can also contribute to directing dissemination strategies and encouraging vaccination through more visual than textual communication in order to reach this population in an efficient way. Nevertheless, there is controversy on the subject, as shown in another state in Brazil where vaccination coverage was better among the children of relatively uneducated mothers, probably due to their need to maintain the child’s vaccination card up-to-date to ensure continuity of their benefits within the Family Benefit Program [25].

No association was found between social class, classified according to family income, and completeness of the vaccination schedule, and this could be a consequence of the homogeneity of income in this population, preventing comparisons between them. There was no family in social class A, a small percentage classified as B and the majority of families belonged to class C and DE with lower incomes. About this, a study conducted in the capital cities of the northeastern states of Brazil showed lower rates of vaccination coverage in children belonging to social class A, i.e. those with the highest family income [26].

No association was found between maternal age and completeness of the vaccination schedule. Nevertheless, a recent publication showed that incomplete vaccination was 26% more common among children of adolescent mothers [7].

In the present study, no association was found between any of the factors related to maternal prenatal care and completeness of the vaccination schedule. This finding contradicts the results of another study showing that maternal prenatal care initiated only in the third trimester of pregnancy and having attended fewer than six prenatal consultations were factors associated with incomplete childhood vaccination [7]. In the present sample, however, almost all the mothers interviewed had received prenatal care, which made comparison between groups impossible.

A limitation of this study is that the evaluation failed to take into account the types of vaccine responsible for the rates of incomplete vaccination schedule, i.e. whether the newer or older vaccines were more likely to be missed. On the other hand, a strongpoint lies in the fact that the data of vaccine administration were obtained directly from the child’s personal health record, thus minimizing the possibility of biases that can occur when data is provided verbally. The fact that an evaluation of internal validity was performed represents another strong point, since this allows the present findings to be compared with results for the same population in different surveys or indeed for the findings to be extrapolated to a population with similar characteristics.

This was the first study to use an epidemiological approach with non-aggregate data at the community level and the results could be proposed as baseline data to monitor completeness of the vaccination schedule in populations living in areas with similar socioeconomic characteristics in Brazil and in other developing countries.

The associated factors identified need to be submitted to a more in-depth analysis by means of a qualitative study focusing on the reasons for incomplete vaccination schedule. Such further evaluation would take into consideration the attitude of the parents or guardians in relation to the child’s vaccination.

## Conclusions

Identification of the factors associated with incomplete vaccination schedule, combined with the monitoring and implementation of an adequate approach strategy by the healthcare teams, could contribute towards achieving the vaccination coverage rates recommended by the national immunization program and avoid the resurgence at epidemic level of infectious diseases in children that are already under control.

## Supplementary information

**Additional file 1.** 2015 National vaccination schedule for children in Brazil.

**Additional file 2.** 2014- Brazil Economic Classification Criteria- Brazil Criterion. Brazilian Association of Research Companies (ABEP-Associação Brasileira de Empresas de Pesquisa)

## Data Availability

The datasets used and/or analysed during the current study are available from the corresponding author upon reasonable request.

## Abbreviations

IMIP: *Instituto de Medicina Integral Professor Fernando Figueira*
UNICEF: United Nations International Children’s Emergency Fund
