# Supplementary material for: Vaccine uptake and associated factors in an irregular urban settlement in northeastern Brazil: a cross-sectional study
Source: BMC Public Health. 2020 Jul 22;20:1152. doi: 10.1186/s12889-020-09247-7 (PMC7376909; doi:10.1186/s12889-020-09247-7)
Supplement: Supplementary file 1 — Additional file 1. 2015 National vaccination schedule for children in Brazil. [file 12889_2020_9247_MOESM1_ESM.docx]

**Additional file 1. 2015 Vaccination schedule for children in Brazil.**

| **Age**  **(months)** | **Vaccines** | | | | | | | | | | |
| --- | --- | --- | --- | --- | --- | --- | --- | --- | --- | --- | --- |
|  | **BCG^a^** | **Poliovirus**  **(OPV/IPV^b^)** | **Pentavalent^c^** | **MMR^d^** | **Hepatitis**  **B** | **10-valent pneumococcal conjugate** | **C**  **meningococcal** | **Rotavirus** | **Hepatitis A** | **DTP^e^** | **Tetraviral^f^** |
| **Birth** | **x** |  |  |  | **x** |  |  |  |  |  |  |
| **1** |  |  |  |  |  |  |  |  |  |  |  |
| **2** |  | **x** | **x** |  |  | **x** |  | **x** |  |  |  |
| **3** |  |  |  |  |  |  | **x** |  |  |  |  |
| **4** |  | **x** | **x** |  |  | **x** |  | **x** |  |  |  |
| **5** |  |  |  |  |  |  | **x** |  |  |  |  |
| **6** |  | **x** | **x** |  |  | **x** |  |  |  |  |  |
| **7** |  |  |  |  |  |  |  |  |  |  |  |
| **8** |  |  |  |  |  |  |  |  |  |  |  |
| **9** |  |  |  |  |  |  |  |  |  |  |  |
| **10** |  |  |  |  |  |  |  |  |  |  |  |
| **11** |  |  |  |  |  |  |  |  |  |  |  |
| **12** |  |  |  | **x** |  | **x** |  |  | **x** |  |  |
| **15** |  | **x** |  |  |  |  | **x** |  |  | **x** | **x** |
| x indicates one dose  ^a^BCG: bacille Calmette-Guérin  ^b^OPV: oral polio vaccine / IPV: inactivated polio vaccine  ^c^Pentavalent: diphtheria–tetanus–pertussis plus Haemophilus influenza type b and Hepatitis B  ^d^MMR: measles-mumps-rubella  ^e^DTP: diphtheria–tetanus–pertussis  ^F^Tetraviral: measles-mumps-rubella-varicella | | | | | | | | | | | |

Adapted from the National Schedule, Ministry of Health, Brazil
