# Supplementary material for: Vaccine uptake and associated factors in an irregular urban settlement in northeastern Brazil: a cross-sectional study
Source: BMC Public Health. 2020 Jul 22;20:1152. doi: 10.1186/s12889-020-09247-7 (PMC7376909; doi:10.1186/s12889-020-09247-7)
Supplement: Supplementary file 2 — Additional file 2. 2014- Brazil Economic Classification Criteria- Brazil Criterion. Brazilian Association of Research Companies (ABEP-Associação Brasileira de Empresas de Pesquisa) [file 12889_2020_9247_MOESM2_ESM.docx]

**Additional file 2: -**2014**-**Brazil Economic Classification Criteria- Brazil Criterion.

Brazilian Association of Research Companies (ABEP-Associação Brasileira de Empresas de Pesquisa)

It is a simplified classification rule for use in research projects whose questionnaire and instructions available at: <http://www.abep.org/criterio-brasil>.

For classification, it uses a points system with variables that include home conditions: bathrooms, domestic servants, possession of goods such as automobiles, microcomputer, dishwasher, refrigerator, freezer, washing machine, DVD, microwave, motorcycle, clothes dryer , education (education level of head of household: Illiterate / incomplete Elementary I; complete Elementary I / incomplete Elementary II; complete Elementary II / incomplete High School; complete High School / incomplete Higher Education; complete Higher Education) and access to public services (running water and paved street). The class profile is home-based.

**Brazil Criteria Cuts**

| Classes | Points |
| --- | --- |
| A1 | 42-46 |
| A2 | 35-41 |
| B1 | 29-34 |
| B2 | 23-28 |
| C1 | 18-22 |
| C2 | 14-17 |
| D | 8-13 |
| E | 0-7 |

**Average Gross family income in the month (R $) by class**

| Economic  Classes | Average gross family income per month in Reais (R $) |
| --- | --- |
| A | 11 037 |
| B1 | 6 006 |
| B2 | 3 118 |
| C1 | 1 865 |
| C2 | 1.277 |
| DE | 895 |

Fonte: LSE 2012 Ibope Media

Classes D and E are scored in the same group for the estimation and construction of samples. The justification for this decision is the small size of class E. as well as class A1. Thus, the average income estimate is made for the class A group.

In the community studied, there were no higher income class A families. Only 15 families were classified as B1 or B2, the majority (72.5%) belonging to class C1 or C2 and 22.6%, DE.
